# Mortality and Survival Rates after Elective Hepatic Surgery in a Low-Volume Centre Are Comparable to Those of High-Volume Centres

**DOI:** 10.5402/2012/783932

**Published:** 2012-07-31

**Authors:** I. E. Nygård, K. Lassen, J. Kjæve, A. Revhaug

**Affiliations:** Department of Digestive Surgery, University Hospital of Northern Norway, University of Tromsø, 9038 Tromsø, Norway

## Abstract

*Background*. Over the last decades, liver resection has become a frequently performed procedure in western countries because of its acceptance as the most effective treatment for patients with selected cases of metastatic tumours. The purpose of this study was to evaluate the results after hepatic resections performed electively in our centre since 1979 and compare the results to those of larger high-volume centres. *Methods*. Medical records of all patients who underwent liver resection from January 1979 to December 2011 were reviewed. Disease-free survival and overall survival were determined by Kaplan-Meier analysis. Risk factors for complications were tested with the log-rank test and the Cox proportional hazard model. Complications were classified according to the modified Clavien classification system. *Results*. 290 elective liver resections were performed between January 1979 and December 2011. There were 171 males (59.0%) and 119 females (41.0%). Median age was 63 years, range 1–87. Overall survival ranged from 0 to 383 months, with a median of 31 months. Five-year survival rate for patients who underwent liver resection for colorectal metastases was 35.8% (34/95). *Discussion*. Hepatic resections are safely performed at a low-volume centre, with regard to perioperative- and in-house mortality and 5-year survival rates.

## 1. Introduction

 Modern liver surgery has developed steadily over the last 30 years. The demonstration of long-term cure after resection of liver metastases from colorectal (C/R) cancer in the 1970's [1] and modern immunological therapies making liver transplantations common procedures [2] constituted the most important driving forces. From a time when liver resections were dramatic and rare procedures, it has become both commonly performed and safe surgery [3–6].

 Advances in surgical planning, operative technique, and perioperative care have resulted in improved short-term outcomes, with experienced centres now reporting in-hospital mortality rates of less than 5%, even with major resections [7–9].

At the University Hospital of Northern Norway (UNN), the first hepatic resection for colorectal cancer metastasis took place in 1979. Since then, the annual number of hepatic resections has increased gradually.

UNN drains a catchment area of almost half a million inhabitants as a tertiary referral centre spread over 113,000 km^2^. Long travelling distances and difficult transport conditions present a necessity for patients to be treated at UNN, in order to maintain effective delivery of health care services. During the 33 year time-period studied, hepatic resections was performed in 1.9 per 100 000 inhabitants per year. The resections have been performed for most types of standard indications of benign and malignant processes.

As modern liver surgery has developed rapidly over the years and our service represents a low-volume centre, it is of outmost importance to ascertain that our results are comparable to the results reported internationally. Accordingly, we have investigated the results of all hepatic resections performed electively in our centre (UNN) since 1979. In particular, we have investigated the results of liver surgery for C/R metastases.

## 2. Material and Methods

Medical records of all patients who underwent liver resection from January 1979 to December 2011 were reviewed. Patients were identified from the Hospital's patient administrative databases, together with information from the Norwegian Central Population Register. A spreadsheet was created for registration of: age, gender, indication for liver resection, type of resection, previous hepatic resections, combined surgery, segments resected, duration of surgery, use of epidural anaesthesia, volume of blood loss, amount and types of fluids received intraoperatively, amount and types of blood product received, use of drains, types of complications, length-of-stay (LOS) until discharge or transferral to primary centre. Death in hospital within 30 days, readmittance within 60 days, months of survival, months of disease-free survival, months until intrahepatic recurrence and months until extrahepatic recurrence were also registered.

Additionally, the records were reviewed for other postoperative events and parameters like fluid, -sodium, and blood products administered until 08:00 A.M. the first postoperative day.

Complications were classified according to the modified Clavien classification system [10].

 Mean blood loss and duration of hospitalisation were compared in early and late time periods, the first period from 1979–1995 and second from 1996–2011, by simply dividing the time period studied in two equal time periods of 17 and 16 years, respectively.

Five-year survival rates were compared by dividing the time-period studied until 2007 into equal time periods, the early period from 1979–1993 and the late period from 1994–2007. In the present study, we defined that a low volume centre conducts less than 20 hepatic resections per year. From 1979–2008, the annual number of hepatic resections was less than 20 per year; the last three years the annual number was more than 20 per year.

30-day survival rate and disease-free survival (months until recurrence) were calculated from the medical records. The records were reviewed between August 2006 and December 2011.

## 3. Statistics

Survival data was obtained from medical records for all patients. Frequencies, descriptive statistics, and crosstabs were calculated for continuous variables and compared with the Mann-Whitney test. The events of death and time to recurrence (disease-free survival) were determined from the Norwegian Central Population Register and medical records. The Kaplan-Meier plots were used to estimate the cumulative incidences of events, and differences in these incidences were evaluated using the log rank test. The Cox proportional hazards regression model using a step-backward-fitting procedure was performed to identify factors that were independently associated with survival, such as age, neoadjuvant chemotherapy, type of resection, blood loss, duration of surgery, complications, and previous resections. All tests were two sided and *P* values of <0.05 were considered to be statistically significant. All statistical analysis was conducted using the Statistical Package for Social Sciences (SPSS) 19.0.

## 4. Results

**Figure 1:**
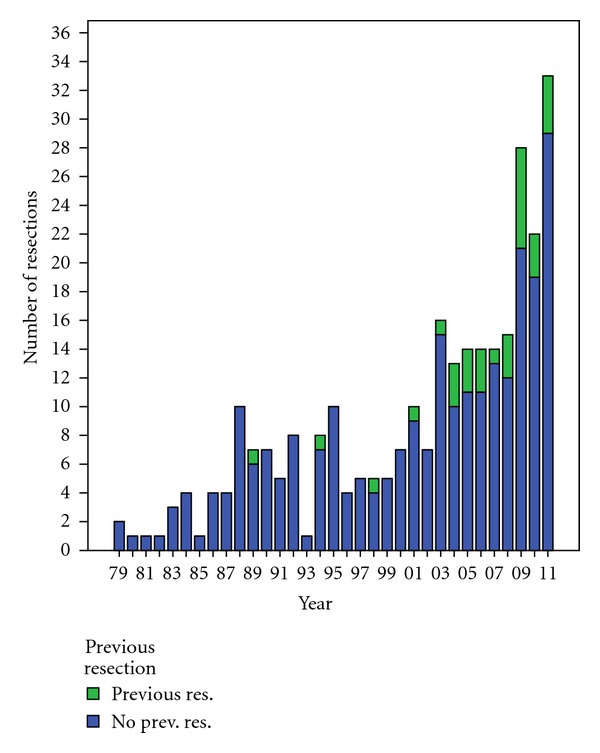


 A total of 290 liver resections were electively performed between January 1979 and December 2011. There were 171 males (59.0%) and 119 females (41.0%). The median age was 63 years, range 1–87 years. The procedures performed were extended right hepatectomies (*n* = 20), extended left hepatectomies (*n* = 3), right hepatectomies (*n* = 52), left hepatectomies (*n* = 20), trisegmentectomies (*n* = 3), bisegmentectomies (*n* = 65), segmentectomies (*n* = 70), and wedge resections (*n* = 57).  Table 1 and Figure 1 show the development of annual resections.

**Figure 2:**
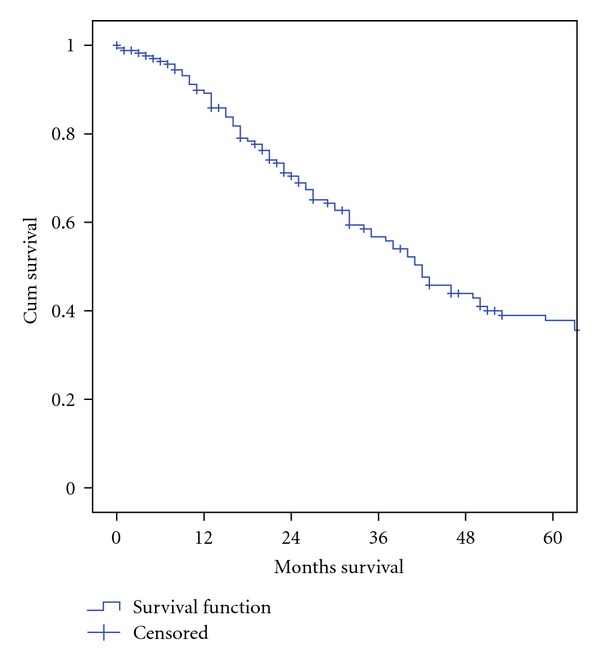


 Colorectal metastasis confined to the liver has been the most frequent indication for hepatic resections (*n* = 174, 60.0%), reflecting the high incidence of cancer in the colon and rectum in our population, Table 2. The five-year survival rate for patients who underwent liver resection for colorectal metastases was 35.8% (34/95), Figure 2. There were 50 (17.2%) metastases from other types of cancer and 20 (6.9%) hepatocellular carcinomas. 26 (9.0%) resections were performed for benign lesions, where hemangioma was the most common pathology.

 Before 2007, a total of 178 liver resections were electively performed, for whom five-year survival could be assessed. Of these, nine patients were operated for neuroendocrine tumours, all of them survived the five-year postoperative period. Eighteen patients were operated with indication hepatocellular carcinoma, and six (33.3%) patients survived five years after surgery. 30 patients were operated for other metastases, and ten of these (33.3%) survived the five-year period. 22 patients had surgery performed for benign tumours, of whom 18 (81.8%) survived the five-year period.

 Of the 290 patients operated at UNN, 109 were transferred to their local hospital for part of the postoperative care. The remaining 181 who were discharged directly from our hospital had a mean length-of-stay (LOS) of 11.1 days. Of these, 78 (43.3%) patients required hospitalisation for less than one week postoperatively while 70 (38.9%) patients required hospitalisation in between one and two weeks postoperatively.

By comparing the early and late time periods, 22.4% and 58.0% required hospitalisation for less than one week postoperatively, while 52.6% and 29.7% required hospitalisation in between one and two weeks after surgery, respectively.

## 5. Postoperative Mortality

 Four out of 290 patients (1.4%) died while still in hospital, all within nine days after surgery. Three patients died before 1996, all from serious cardiovascular complications. One patient operated in 1983 for colorectal cancer metastases lost 7000 mL blood during surgery and suffered a myocardial infarction intraoperatively. Another patient operated in 1986 for hepatocellular carcinoma lost 2500 mL blood during surgery. The patient had myocardial fibrosis that could have been caused by chemotherapy and atherosclerotic coronary vessels and died nine days postoperatively. The third patient was operated in 1995 for colorectal cancer metastases lost 19500 mL blood during surgery and died from acute cardiac failure intraoperatively. The fourth patient was operated in 2011 for hepatocellular carcinoma lost 250 mL blood during surgery and died five days postoperatively due to a severe pneumonia.

## 6. Postoperative Complications

Out of 290 patients, 65 (22.4%) developed complications, and six of these patients developed more than one complication. All complications were classified according to the modified Clavien classification system, Table 3.

 The most common complication was pneumonia, which occurred in seventeen patients (5.9%). Other complications were wound infections in five patients (1.7%), serious cardiovascular complications in three patients (1.0%), such as myocardial infarction on the first postoperative day in one patient, perioperative heart failure in another patient, and myocardial fibroses and coronary atherosclerosis in the third of these patients.

**Figure 3:**
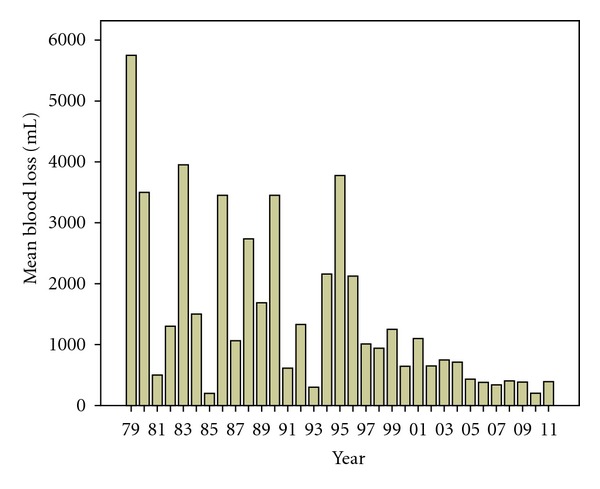


 Blood loss during elective surgery showed a clear reduction through the time period studied. It varied from 0 to 19500 mL through the whole period. In 1979, the mean blood loss for the two patients operated was 5750 mL, while in 2011 the mean blood loss was 392 mL, Figure 3.

## 7. Postoperative Survival

 Of 290 patients who underwent liver resection over the last 33 years, 134 (46.2%) were alive by December 2011. Overall survival ranged from 0 to 383 months, with a median of 31 months. The actual 2-year survival was 71.5% (168 of 235) for all patients operated on >2 years ago. The actual 5-year survival was 45.5% (81 of 178) for all patients operated on >5 years ago.

The five-year survival rate for patients who underwent liver resection for C/R metastases was 35.8% (34/95). The two-year survival rate was 71.1% (96/135). Of patients who survived five year postoperatively, 27 of 34 (79.4%) were considered cured five years after surgery. Of patients that died during five-year control periods, 51 of 61 patients (83.6%) had recurrence of metastasis. By dividing time in early and late periods, we found a five-year survival of 42.3% (11/26) from 1979–1993. The late period, from 1994–2007, had a five-year survival of 33.3% (23/69).

 Five-year survival rate for patients who lost more than one litre blood during surgery was 35.1% (20/57) and for those who lost less than one litre 49.6% (58/117) (*P* = 0.002).

## 8. Discussion

This report presents a complete single-centre cohort from the first elective liver resection performed in 1979, totalling 290 resections during 33 years. The main objective was to investigate the postoperative survival rate after all elective hepatic resections at our centre and to compare the results to those of larger high-volume centres. The perioperative mortality rate after all resections was 1.4%, which is comparable to the results of this surgery reported internationally with in-hospital mortality rates of less than 5% [9].

Three out of four perioperative deaths occurred before 1996, one patient died in 2011 due to a severe pneumonia. A recent study from a high-volume centre is now reporting zero operative mortality for eight years [4]. Despite the low volume in our hospital, our liver resection mortality has followed the international development for hepatic surgery.

Hepatic resections were previously performed in a selected group of patients with limited indications and only resections with a curative intention were performed. For patients operated from 1979–1983, the five-year survival rate was 55.6% (5/9). International guidelines are now considering hepatic resection as both curative and palliative treatment. This includes patients with multiple metastases, resulting in a five-year survival rate of 33.3% (15/45) for patients operated from 1999–2003.

Liver metastasis from colorectal cancer was the most common pathology (*n* = 174). The five-year survival rate for patients who underwent liver resection for colorectal metastases was 35.8% (34/95) which is comparable to recent studies from high-volume centres reporting five-year survival rates of 29–37% [3, 7]. Resections were performed in 1.9 per 100 000 inhabitants per year which also is in accordance with the prevalence in similar populations in western countries.

There has been a significant reduction in operating time, total amount of blood loss and 30 days mortality after surgery. A reduction in intraoperative bleeding and a reduction in blood transfusions indicates better surgical techniques during the latest operation period.

At our hospital there are few surgeons performing liver resections. To justify the activity in a small centre, the few surgeons performing hepatic surgery have regularly been taking part in international courses and spending long periods (months and years) in high-volume centres around the world.

A parallel research program of experimental surgery related to liver failure and liver regeneration has also added importantly to the maintenance of updated knowledge of liver pathophysiology as well as technical skills amongst the involved personnel. More than 700 large animals have been used in such research in our centre over the last 10 years, resulting in several theses and more than 20 peer-reviewed publications from our Department.

A significant reduction in hospital stay after liver surgery has been demonstrated in recent years. This is a result of gradually implementing an enhanced recovery after surgery protocol (ERAS) for our liver-resected patients [11] but also due to the high rate of minor resections performed in late periods.

## 9. Conclusion

Perioperative and in-hospital mortality as well as 5-year survival rates are comparable to those of from high-volume centres. By following international guidelines, major hepatic resection for malignant or benign disease can be performed safely with minimal morbidity and mortality at a low-volume centre.

## Figures and Tables

**Table 1:** Operative procedures.

	Frequency	Percent
Wedge	57	19.7
Segmentectomy	70	24.1
Bisegmentectomy	65	22.4
Trisegmentectomy	3	1.0
Right hepatectomy	52	17.9
Left hepatectomy	20	6.9
Extended right hepatectomy	20	6.9
Extended left hepatectomy	3	1.0

Total	290	100.0

**Table 2:** Indications.

	Frequency	Percent
Liver metastasis from colorectal cancer	174	60.0
Neuroendocrine carcinoid	16	5.5
Hepatocellular carcinoma	20	6.9
Other metastasis/cancer	50	17.2
Benign tumors	26	9.0
Trauma	4	1.4

Total	290	100.0

**Table 3:** Complications of 290 liver resections classified according the Clavien system.

Grades	Complications	Frequency
Grade 1 (*n* = 5; 1.7%)	Dyspnoea	1
	Mild pleural effusion treated conservatively	4
Grade 2 (*n* = 22; 7.6%)	Pneumonia requiring antibiotics	17
	Wound infection requiring antibiotics	5
Grade 3(a) (*n* = 2; 0.7%)	Pleural effusion requiring thoracic drainage	1
	Gastrointestinal complications treated conservatively	1
Grade 3(b) (*n* = 35; 12.1%)	Postoperative surgical bleeding requiring relaparotomy	7
	Intraabdominal abscess/biloma requiring surgery	14
	Wound rupture/wound infection requiring surgery	3
	Gastrointestinal complications requiring relaparotomy (ileus, anastomotic leakage)	11
Grade 4(a) (*n* = 6; 2.1%)	Liver failure	3
	Serious cardiovascular complications	3
Grade 4(b) (*n* = 1; 0.3%)	Systemic Inflammatory Response Syndrome/Sepsis	1
Grade 5 (*n* = 4; 1.4%)	Death within 30 days after surgery	4
